# Evidence of Clonal Expansion in the Genome of a Multidrug-Resistant *Mycobacterium tuberculosis* Clinical Isolate from Peru

**DOI:** 10.1128/genomeA.00089-14

**Published:** 2014-02-27

**Authors:** M. Galarza, D. Tarazona, V. Borda, J. C. Agapito, H. Guio

**Affiliations:** a Laboratorio de Biotecnología y Biología Molecular, Instituto Nacional de Salud, Lima, Peru; b Asociación Latinoamericana de Biotecnología (ALBIOTEC), Lima, Peru; c Unidad de Biotecnología Molecular-Laboratorios de Investigación y Desarrollo de Ciência y Tecnología (LID), Universidad Peruana Cayetano Heredia, Lima, Peru

## Abstract

We report the genome sequence of *Mycobacterium tuberculosis* INS-MDR from Peru, a multidrug-resistant tuberculosis (MDR-TB) and Latin American-Mediterranean (LAM) lineage strain. Our analysis showed mutations related to drug resistance in the *rpoB* (D516V), *katG* (S315T), *kasA* (G269S), and *pncA* (Q10R) genes. Our evidence suggests that INS-MDR may be a clonal expansion related to the African strain KZN 1435.

## GENOME ANNOUNCEMENT

The emergence of multidrug-resistant (MDR) strains for tuberculosis (TB) is resulting in one of the major worldwide public health problems. In 2012, the incidence rate for TB in Peru was 95 cases/100,000 population, of which 3.9% were new cases of MDR-TB (1), and from these MDR-TB cases, 79% were from Lima (2). Currently, there is no genomic information on an MDR strain from Peru. Thus, obtaining this information is a key step in understanding the biology of the pathogen and improving treatment for TB (3). Here, we report genomic features of a multidrug-resistant strain of *Mycobacterium tuberculosis*, INS-MDR, from a patient with active TB from Peru.

The establishment of this strain’s lineage was based on 24 mycobacterial interspersed repetitive unit-variable number of tandem repeat (MIRU-VNTR) loci (4) and on single-nucleotide polymorphisms (SNPs) based on phylogeny (5). The genome sequencing of INS-MDR was performed using an Illumina HiSeq 2000 sequencer with coverage of 1,331×. Resulting paired-end reads were assembled with BWA v 0.5.9-r16 (6), using the H37Rv genome (NC000962.3) as a reference. The genomic sequence was annotated with the RAST server (7), Prokaryotic Genome Annotation Pipeline (PGAAP), and Clusters of Orthologous Groups (COG) (8) databases. A comparative analysis was carried out between INS-MDR and KZN 1435, an MDR strain from South Africa (9), using SNPsFinder (10) to identify the differences in intergenic coding regions and gene ontology classifications (COG).

It has been determined that INS-MDR belongs to the LAM lineage. We obtained 58,157,302 paired-end reads that, after the assembly, resulted in 22 contigs comprising about 99.98% compared to the H37Rv genome. INS-MDR is 4,383,671 bp long, with an average GC content of 65.6%.

A total of 805 polymorphisms with respect to H37Rv were observed, with 703 of these located in the coding regions that were classified in COG categories as follows: secondary metabolite biosynthesis, transport, and catabolism (*n* = 38); lipid transport and metabolism (*n* = 41); replication, recombination, and repair (*n* = 32); energy production and conversion (*n* = 35); amino acid transport and metabolism (*n* = 30); carbohydrate transport and metabolism (*n* = 27); cell motility (*n* = 21); cell wall/membrane/envelope biogenesis (M) (*n* = 23); coenzyme transport and metabolism (*n* = 22); signal transduction mechanisms (*n* = 22); inorganic ion transport and metabolism (*n* = 22); transcription (*n* = 20); translation, ribosomal structure, and biogenesis (*n* = 16); posttranslational modification and protein turnover (*n* = 13); nucleotide transport and metabolism (*n* = 11); defense mechanisms (*n* = 5); cell cycle control, cell division, and chromosome partitioning (*n* = 9); and RNA processing and modification (*n* = 2).

We identified a mutation on the *rpoB* gene (D516V) related to resistance to rifampin (11), mutations on the *katG* (S315T) and *kasA* genes (G269S) related to resistance to isoniazid (12, 13), and a mutation on the *pncA* gene (Q10R) related to pyrazinamide resistance (14). Also, we found mutations on the *gyrA* gene (E21Q, S95T, G247S, G668D) and a mutation on the *embB* gene (Y319S) that do not confer resistance (15–17).

Comparison of the KZN 1435 and INS-MDR strains demonstrated that, despite the differences in geographic origin and the high incidence of TB in South Africa (1), the strains have similar proportions of SNPs, showing high degrees of conservation of genome structure. These results suggest that the outbreak of drug-resistant tuberculosis in Lima may be a clonal expansion of the same strain; however, more genomic information is required.

### Nucleotide sequence accession numbers.

This whole-genome shotgun project has been deposited at DDBJ/EMBL/GenBank under the accession number JAQI00000000. The version described in this paper is version JAQI01000000.
